# Strategies of *Vibrio parahaemolyticus* to acquire nutritional iron during host colonization

**DOI:** 10.3389/fmicb.2015.00702

**Published:** 2015-07-09

**Authors:** Nidia León-Sicairos, Uriel A. Angulo-Zamudio, Mireya de la Garza, Jorge Velázquez-Román, Héctor M. Flores-Villaseñor, Adrian Canizalez-Román

**Affiliations:** ^1^Unidad de Investigación, Facultad de Medicina, Universidad Autónoma de SinaloaCuliacán, Mexico; ^2^Departamento de Investigación, Hospital Pediátrico de Sinaloa “Dr. Rigoberto Aguilar Pico”Culiacán, Mexico; ^3^Maestría en Ciencias de la Salud, Facultad de Medicina, Universidad Autónoma de SinaloaCuliacán, Mexico; ^4^Departamento de Biología Celular, Centro de Investigación y de Estudios Avanzados del Instituto Politécnico NacionalMexico, Mexico

**Keywords:** *Vibrio parahaemolyticus*, iron, virulence, host iron proteins, mechanism of acquisition

## Abstract

Iron is an essential element for the growth and development of virtually all living organisms. As iron acquisition is critical for the pathogenesis, a host defense strategy during infection is to sequester iron to restrict the growth of invading pathogens. To counteract this strategy, bacteria such as *Vibrio parahaemolyticus* have adapted to such an environment by developing mechanisms to obtain iron from human hosts. This review focuses on the multiple strategies employed by *V. parahaemolyticus* to obtain nutritional iron from host sources. In these strategies are included the use of siderophores and xenosiderophores, proteases and iron-protein receptor. The host sources used by *V. parahaemolyticus* are the iron-containing proteins transferrin, hemoglobin, and hemin. The implications of iron acquisition systems in the virulence of *V. parahaemolyticus* are also discussed.

## Introduction

*Vibrio parahaemolyticus* is a halophilic Gram-negative bacterium that naturally inhabits marine and estuarine environments (Gavilan et al., 2013). The more recent distribution of this species has been driven by climatic conditions and the eutrophication of regional waters throughout the world (Baumann et al., 1984). *V. parahaemolyticus* can survive in a wide variety of niches in a free-swimming state, and its motility is conferred by a single polar flagellum. Alternatively, this bacterium can be found in a sessile state, attached to inert or animate surfaces, such as suspended particulates, zooplankton, fish, and shell-fish (McCarter, 1999). Many strains of *V. parahaemolyticus* are strictly environmental, though small subpopulations can be opportunistic pathogens that may cause gastroenteritis, wound infection, and septicemia (Joseph et al., 1982; McCarter, 1999). As with all bacteria, *V. parahaemolyticus* reproduces via binary fission without a systematic exchange of genes with other individuals of the same species, leading to essentially clonal reproduction (Garcia et al., 2013); however, only some of these clones cause diarrhea in humans. Strains of *V. parahaemolyticus* belong to different serogroups and can produce a number of different lipopolysaccharide (O) and capsular (K) antigens, constituting the primary basis for strain classification (Nair et al., 2007; Garcia et al., 2013).

Since its discovery in 1950 by Tsunesaburo Fujino of Osaka University after an outbreak due to the ingestion of contaminated seafood, *V. parahaemolyticus* has been recognized as a leading cause of intestinal infection throughout coastal countries worldwide (Fujino et al., 1953). Following the consumption of raw or undercooked seafood, individuals infected with virulent strains may present clinical symptoms that include diarrhea, vomiting, nausea, abdominal cramping, and low-grade fever. Exposure to *V. parahaemolyticus* can also lead to wound infection and septicemia in certain medical conditions, such as immune deficiency (Su and Liu, 2007).

The infections and outbreaks caused by *V. parahaemolyticus* prior to 1996 were geographically isolated and associated with a diversity of serotypes (Wong et al., 2000; Chowdhury et al., 2004). However, the later increase in outbreaks was linked to the occurrence of gastroenteritis throughout Asia associated with a new and unique clone of serotype O3:K6 that emerged in Kolkata, India, in 1996 (Matsumoto et al., 2000; Ansede-Bermejo et al., 2010; Pazhani et al., 2014). This clone rapidly spread throughout the majority of Southeast Asian countries in the same year (Okuda et al., 1997b; Ansede-Bermejo et al., 2010; Gavilan et al., 2013). This clone, referred to as the pandemic clone, has since spread globally from Southeast Asia to Europe, Africa, and the Americas (Okuda et al., 1997a; Nair et al., 2007; Paranjpye et al., 2012; Yano et al., 2014). In particular, some countries on the American continent have reported cases of gastroenteritis due to the pandemic O3:K6 strain and its serovariants; the pandemic strain was first detected in Peru, subsequently spread to Chile in 1998, to the U. S. in the same year, to Brazil in 2001 and to Mexico in 2004 (Velazquez-Roman et al., 2012, 2013). Based on various molecular methods, these widespread pandemic O3:K6 strains have been found to be genetically closely related and appear to constitute a clone that differs significantly from the non-pandemic O3:K6 strains that were isolated prior to 1996 (Okuda et al., 1997b; Wong et al., 2000; Lan et al., 2009), with several serovariants apparently emerging since 1996 (Nair et al., 2007; Velazquez-Roman et al., 2012, 2013; Al-Othrubi et al., 2014; Pazhani et al., 2014; Letchumanan et al., 2015). The global occurrence of *V. parahaemolyticus* strains emphasizes the importance of understanding their many virulence factors as well as the mechanisms used to acquire nutrients from the environment and the effects on human hosts.

*Vibrio parahaemolyticus* possesses a wide variety of virulence factors that cause damage such as adhesins, toxins, and secreted effectors involved in attachment, cytotoxicity, and enterotoxicity (Broberg et al., 2011; Zhang and Orth, 2013). The two main factors are a thermostable direct hemolysin (TDH) and a thermostable direct hemolysin-related hemolysin (TRH) encoded by the *tdh* and *trh* genes, respectively (Honda et al., 1982; Nishibuchi et al., 1992; Vongxay et al., 2008). Nonetheless, the isolation of *V. parahaemolyticus* strains lacking functional *tdh* and *trh* genes from human infection cases and the analysis of the genome sequence of *V. parahaemolyticus* strain RIMD2210633 suggest that other virulence factors also play a role in the disease caused by this bacterium (Makino et al., 2003; Okada et al., 2009). The genome of *V. parahaemolyticus* contains two sets of type III secretion system (T3SS) gene clusters that function in the secretion and translocation of virulence factors into eukaryotic cells. These appear on each of the two chromosomes (Makino et al., 2003; Caburlotto et al., 2009). T3SSs utilize a needle-like apparatus to translocate into host cells effectors that target and hijack multiple eukaryotic signaling pathways. Indeed, T3SSs are essential virulence machines used by numerous bacterial pathogens, including *Yersinia, Salmonella, Shigella*, and pathogenic *Escherichia coli* (Makino et al., 2003).

Other systems or mechanisms that play an important role as virulence factors in *Vibrio* and all pathogenic microorganisms are those that confer the ability to acquire nutrients from the environment in which they live. These systems ensure that pathogens successfully reproduce and become established in a host. For example, the capacity to acquire nutrients such as iron (Fe) from a host is an ability obtained by pathogenic microorganisms during evolution. In fact, it has been speculated that the evolutionary pressure for microbes to develop pathogenic characteristics was to access the nutrient resources supplied by animals (Tanabe et al., 2012). The environment inside the colonized host has led to the evolution of new bacterial characteristics to maximize such new nutritional opportunities (Rohmer et al., 2011; Tanabe et al., 2012).

Currently, access to host nutrients is regarded as a fundamental aspect of an infectious disease. During the invasion of the human host, pathogens encounter complex nutritional microenvironments that could change, for example; the increase in inflammatory response due to the infection, local hypoxia in some tissues (Payne, 1993; Cassat and Skaar, 2013). The host can limit microbial access to nutrient supplies as a defense mechanism against the pathogens, however, the pathogens can counteract this by developing metabolic adaptations or improved mechanisms of nutrient acquisition to successfully exploit available host nutrients for their proliferation (Payne, 1993). Recent studies have pointed out an emerging paradigm that has been designated as ‘nutritional virulence’ (Abu Kwaik and Bumann, 2013). Although this term is applied to the acquisition of amino acids and carbon sources, certain nutritional ions or metals that are essential for cellular growth and other metabolic processes could be considered as part of this paradigm. As one of the most fundamental aspects of infectious diseases is the microbial acquisition of nutrients *in vivo*, which positively impacts in virulence as well as antibiotic resistance (Santic and Abu Kwaik, 2013), we suggest that the process of iron acquisition systems used by pathogenic microorganisms may be considered in the concept of ‘nutritional virulence.’

Iron (Fe) is an essential element for almost all cells, including most bacteria because it serves as a cofactor for metabolic processes, such as redox reactions, nucleic acid synthesis, and electron transfer (Tanabe et al., 2012). Iron is the fourth most abundant element on the Earth’s crust. In nature, there are two states of iron: (1) ferrous iron (Fe^2+^), which is toxic because it leads to the production of hazardous reactive oxygen species (ROS), including superoxide, in the presence of oxygen; and (2) ferric iron (Fe^3+^), which is insoluble under normal physiological conditions. Fe is bound to ligands, primarily proteins, in iron-dependent organisms, and trace Fe concentrations are necessary for all organisms, ranging between 0.4 and 4 μM in the majority of both eukaryotic and prokaryotic cells (Weinberg, 1974). However, there are bacteria such as some lactobacilli that are iron independent because they utilize manganese (Mn) and other cations as cofactors in their enzymes (Imbert and Blondeau, 1998).

The pathogenic microorganisms that infect mammalian hosts encounter diverse and changing environments. For example; the pH within the human body is usually neutral (7.4), but it can range from 1.0 in the stomach to 8.0 in urine. Also, if they move deeper into host tissues at mucosal surfaces, such as those from the lumen, the multilamellar mucus, and the epithelial cells of the stomach, pathogens confront drastically different or hostile environments. Some mucosal surfaces are well oxygenated, but others possess areas of low oxygen tension, for example the oral cavity, large intestine, female genital tract, abscesses and damaged tissues (Rohmer et al., 2011). The level of free Fe in mammalian bodily fluids is variable (∼10^-18^ M) but always far below the concentrations required for optimal bacterial growth (10^-6^ M), causing bacteria to rely on their own strategies or mechanisms for obtaining this metal. In an infected site there are numerous physiologically specialized environments that bacteria might encounter or colonize. For example, within the small intestine, there are variable conditions different from those found between caecum and colon (Rohmer et al., 2011). For all these reasons mentioned above, pathogens move through multiple diverse environments throughout their life cycle, and to accomplish this they require the regulation, coordination, and utilization of multiple bacterial metabolic pathways. Bacteria often use metabolic cues in order to regulate their metabolism and virulence functions to be successful as pathogens (Rohmer et al., 2011). Because they depend upon Fe as a vital cofactor that enables a wide range of key metabolic activities, bacteria must therefore ensure a balanced supply of this essential metal; accordingly, they invest considerable resources into its acquisition and employ elaborate control mechanisms to alleviate both iron-induced toxicity and Fe deficiency (Rangel et al., 2008).

The Fe concentration in coastal waters ranges from 1.3–35.9 nM up to 23.1–573.2 nM (Gledhill and Buck, 2012), a concentration that may be sufficient to support the growth of *V. parahaemolyticus*; however, *V. parahaemolyticus* also infects humans. The human body contains 3.8 g (in men) and 2.3 g (in women) of Fe. 20 mg of Fe is required daily for the production of hemoglobin (Hb) for new erythrocytes in order to preserve Fe homeostasis. Iron absorption from the diet, however, supplies only 1–2 mg daily, and the remaining Fe is derived by recycling Fe from senescent red blood cells (Ganz and Nemeth, 2006). Most bodily Fe is found in heme proteins (Hb, myoglobin, cytochromes, and multiple enzymes), and the second largest Fe pool is found in ferritin (also in hemosiderin). The remaining Fe is found in other proteins, such as iron-sulfur cluster enzymes, Fe-chelating proteins (Tf and Lf), and a pool of accessible Fe ions called the labile Fe pool (LIP), all of them constitutes the iron-containing proteins involved in metabolic pathways from hosts.

**FIGURE 1:**
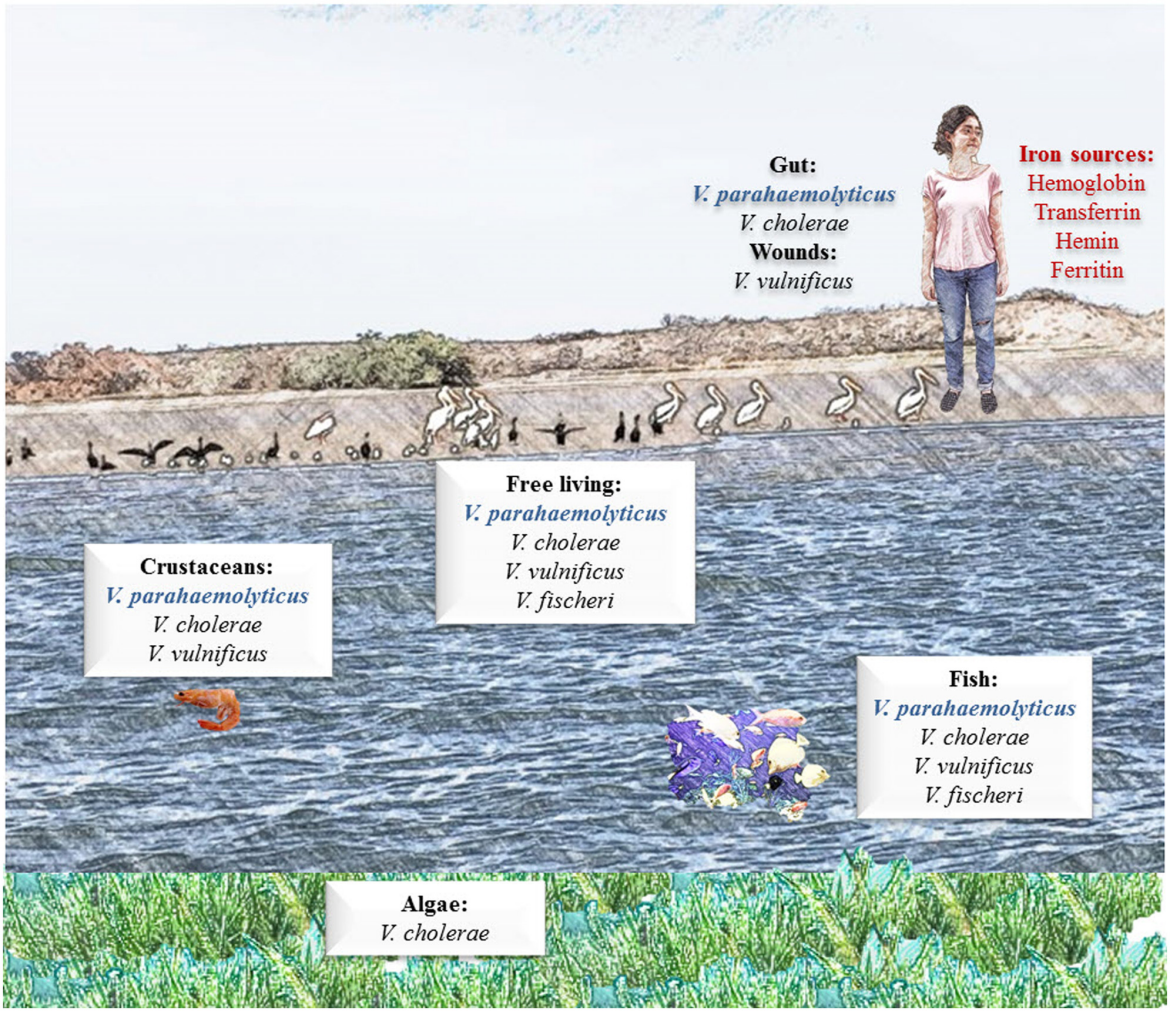
**Iron sources available in the environmental niches of *Vibrio parahaemolyticus*.** *V. parahaemolyticus* is an obligate halophilic organism, meaning that it requires salt to live. This organism is naturally occurring and found worldwide. It can commonly be found free swimming or attached to underwater surfaces and is found at high concentrations in areas of significant seafood consumption. Humans can acquire *V. parahaemolyticus* infection from infected seafood; once infected, established *V. parahaemolyticus* can acquire iron from different iron-containing proteins, such as hemoglobin (Hb) and transferrin (Tf).

Inside the human body the solubility of iron is extremely low, because the Fe exists in insoluble mineral complexes, or under aerobic, aqueous, and neutral pH conditions, that difficult the access of bacteria to this element. Besides, Fe is bound to mammalian high-affinity iron-binding proteins such as Tf, Lf, and Ft and in consequence, many bacteria have developed high-affinity Fe transport systems to acquire Fe from sources in their niches (Rangel et al., 2008; Jin et al., 2009; Tanabe et al., 2012). The Fe sources available in the different environmental niches of *V. parahaemolyticus* are described and discussed in **Figure 1**.

## Role of Iron in the Virulence of *Vibrio parahaemolyticus*

Iron regulates virulence factors of *V. parahaemolyticus* and almost all pathogenic bacteria. Inside a host the Fe concentration is very low, so many pathogens uses this (low-iron conditions) for inducing expression of genes involved in the virulence (Litwin and Calderwood, 1993). The presence of ferric Fe in bacterial growth media has been found to increase the adherence intensities of virulent *V. parahaemolyticus* strains to human fetal intestinal (HFI) cells *in vitro* (Hackney et al., 1980). Intraperitoneally injection with *V. parahaemolyticus* in the presence of ferric ammonium citrate in mice increased the bacterial proliferation, thus enhancing the lethality toward infected mice. *V. parahaemolyticus* cultures in low-iron conditions showed better proliferation than iron-rich cultures in response to the addition of supplementary Fe. Also, the production of thermostable direct toxin (TDH) by the hemolytic strains of *V. parahaemolyticus* was higher in iron-limited cultures than in iron-rich cultures, though the production of TDH by both iron-limited and iron-rich cultures was inhibited by the addition of Fe. In conclusion, the enhancement of *V. parahaemolyticus* virulence in the model mice likely occurred through the increase of bacterial proliferation *in vivo* and not the stimulation of TDH production. The *V. parahaemolyticus* precultured under iron-limited conditions may be more adaptable to the *in vivo* environment (Wong and Lee, 1994; Funahashi et al., 2003; Gode-Potratz et al., 2010).

The effect of lysed blood on the virulence of *V. parahaemolyticus* in mice was also investigated, and a factor released by erythrocyte lysis was found to greatly reduce the 50% lethal dose of *V. parahaemolyticus* in mice. Similar effects were observed with ferric ammonium citrate and Mn sulfate. Authors conclude that Fe from the lysed blood is involved in the virulence of *V. parahaemolyticus* (Karunasagar et al., 1984).

In a recent work, Gode-Potratz et al. (2010) demonstrated that metal ions play distinct roles in modulating gene expression and behavior in *V. parahaemolyticus*. In this work, high-calcium and low-iron growth conditions stimulated the induction of swarming and T3SS regulons from *V. parahaemolyticus* (Gode-Potratz et al., 2010). Swarming is a particular adaptation of many bacteria to grow in surfaces. In *V. parahaemolyticus* swarming is done by lateral flagella that enable the bacteria move over and colonize surfaces (Gode-Potratz et al., 2010). The authors concluded that swarming plays a signaling role with global consequences on the regulation of gene sets that are relevant for surface colonization and infection and that stimulation depends on the level of Fe present in the environment (Gode-Potratz et al., 2010).

Iron also regulates virulence factors in other members of the genus *Vibrio*. Based on the description of the virulence-enhancing effect of ferric ammonium citrate on *V. cholerae* by Joo (1975), other work has supported the role and importance of Fe in the virulence of *Vibrio* sp. (Joo, 1975). It has been demonstrated that the iron overload in humans increases the virulence of pathogens *in vivo*. For example in patients with liver diseases and hemochromatosis, iron overload states are common. It is known that these conditions predispose patients to recurrent infections, septicemia, and high mortality due to pathogens such as *V. vulnificus* (Bullen et al., 1991; Barton and Acton, 2009). This was corroborated by injecting mice with *V. vulnificus* and Fe, resulting in a lower 50% lethal dose and in a reduction in the time to death post-infection (Wright et al., 1981). In addition, elevated serum Fe levels were also produced by liver damage due to injections. Because the infections with *V. vulnificus* are acquired through the consumption of contaminated seafood, the role of iron on infections acquired by the oral route was also studied in mice. On the other hand, the role of iron on the growth of *V. vulnificus* in human and rabbit sera with injections of Fe or Tf were also studied. In the results, lethality and growth of *V. vulnificus* were more efficient in rabbit and human sera; respectively (Wright et al., 1981). Next, an induced peritonitis model was employed in mice to determine whether heme-containing molecules enhance the lethality of infections of *V. vulnificus* (Helms et al., 1984). In this model, the lethality toward mice inoculated intraperitonally with the bacteria and treated with methemoglobin, or hematin but not by myoglobin, was increased compared with those untreated (Helms et al., 1984). These results indicated that *V. vulnificus* has the capacity to produce fatal human infections because of its ability to use human proteins that bind Fe (Helms et al., 1984). Additionally, *V. vulnificus* strains isolated from different sources of Cuddalore coastal waters were tested for their virulence activity based on their LD50 values in mice. The LD50 was in the range of 10^4^–10^7^ cells in normal mice, but 10^1^–10^2^ cells in iron-injected mice, thus reinforcing the idea that Fe (acquired from human sources *in viv*o) may play a major role in the pathogenesis of *V. vulnificus* (Jayalakshmi and Venugopalan, 1992). Additionally, the virulence mechanisms of *V. vulnificus* biotype 1 and biotype 2 were studied and compared in mice. Both strains presented several properties in common, including capsule expression, the uptake of various Fe sources, and the production of exoproteins (Wright et al., 1981; Amaro et al., 1994). Taken together, data support the importance of iron in the pathogenesis of *Vibrio* sp. and *V. parahaemoluticus in vivo* and *in vitro.*

## Iron regulatory Proteins and Mechanisms in *V. parahaemolyticus*

The study of Fe acquisition systems in *E. coli* led to the discovery of Fur, a DNA binding protein of 16.8 kDa, product of the *fur* (ferric uptake regulation) gene, that represses the transcription of genes involved in Fe uptake systems in iron replete conditions (Litwin and Calderwood, 1993). When the intracellular Fe concentration increases, Fur forms a dimer together with ferrous Fe (Fe^2+^) and binds to a consensus sequence (Fur-box), which overlaps the promoters of Fur-target genes, resulting in the inhibition of transcription. Although the role of Fur as a repressor is well-documented, emerging evidence demonstrates that Fur can function as an activator (Troxell and Hassan, 2013). Additionally to *E. coli*, Fur has been identified in other Gram-negative and Gram-positive bacteria. An interesting finding was that Fur also participates in functions different to the Fe metabolism for example; defense against oxygen radicals, metabolic pathways, bioluminescence, chemotaxis, swarming and production of toxins, and other virulence factors (Litwin and Calderwood, 1993). We can speculate about the importance of these Fe dependent mechanisms for bacterial virulence inside a host *in vivo*.

*Vibrio parahaemolyticus* contains a Fur protein that is 81% identical with the Fur protein from *E. coli* and over 90% identical with those of the *Vibrio* sp. (Yamamoto et al., 1997). Funahashi et al. (2002) reported that *V. parahaemo*lyticus *psuA* and *pvuA* genes (which encode the TonB-dependent outer membrane receptors for a putative ferric siderophore and ferric-vibrioferrin), are regulated by Fur (Funahashi et al., 2002). Additionally, a homolog of the *iutA* gene in *V. parahaemolyticus* (which encodes for the receptor of ferric aerobactin) is apparently regulated by Fur (Funahashi et al., 2003). Furthermore, Fur regulates *V. parahaemolyticus peuA* gene (which encodes for an alternative ferric-enterobactin receptor; Tanabe et al., 2014).

Fur has been involved in the regulation of other virulence factors from *Vibrio* sp. Lee et al. (2013) demonstrated that in *V. vulnificus* Fur regulates hemolysin production at the transcriptional (vvhBA operon) and post-translational (by regulating the expression of two VvhA-degrading exoproteases, VvpE, and VvpM) levels (Lee et al., 2013). In contrast, other transcriptional regulators such as AraC-type family members and LysR-type family members, have been shown to activate transcription initiation of genes involved in the synthesis and utilization of siderophores in bacteria (Balado et al., 2009; Tanabe et al., 2012). In *V. cholerae* Fur regulates the expression of a number of genes in response to changes in the level of available iron. Fur usually acts as a repressor, but it has been shown that Fur positively regulates the expression of ompT, which encodes a major outer membrane porin, involved in the virulence of *V. cholerae* (Craig et al., 2011). It has been reported that Fur also represses the synthesis of RyhB, which negatively regulates genes for iron-containing proteins involved in the tricarboxylic acid cycle and respiration as well as genes for motility and chemotaxis (Wyckoff et al., 2007). Mey et al. (2005) reported the effects of iron and Fur on gene expression in *V. cholerae*. According with this work, nearly all of the known iron acquisition genes were repressed by Fur under iron-replete conditions, and also those genes involved in the transport of iron inside of pathogens (Mey et al., 2005). The iron transport systems regulated negatively by Fur in iron-replete conditions were *feo* and *fbp* genes (involved in the transport of ferrous and ferric iron inside cells; respectively). Both were found to be negatively regulated by iron and Fur (Mey et al., 2005). This is consistent with others genes involved in iron acquisition; in high concentrations of this nutrient the genes for iron acquisition systems are repressed.

## Iron acquisition Systems Used by Pathogenic Microorganisms

An obligate question is how does *V. parahaemolyticus* acquire iron? This theme is complex; however, it has been well established in other pathogens. To acquire Fe from host sources, microorganisms generally use the iron-acquisition systems described below.

### Receptors for Host Iron-Containing Proteins

Transferrins (Tfs) are a family of iron-binding glycoproteins that chelate free ferric Fe in biological fluids (Crichton and Charloteaux-Wauters, 1987). Bacteria such as *Neisseria gonorrhoeae* and *Haemophilus influenzae* are able to use the Fe in these proteins by binding Tf and Lf iron directly through the use of membrane receptors or binding proteins for host iron-glycoproteins (Tbp and Lbp, respectively). These receptors have been reported to be induced in some bacteria in the absence of Fe (Gray-Owen and Schryvers, 1996). Some receptors are specific for one iron-containing protein. On the contrary, other receptors that recognizes Tf, also can recognize Lf or other iron proteins, these receptors have been found in *Neisseria* (Morgenthau et al., 2013). Tf binding protein (TbpA) and lactoferrin binding protein (LbpA) are receptors that share 25% amino acid identity and a high degree of similarity. These Tf and Lf receptors can be promiscuous; for example, TbpA can recognizes Lf in some bacteria. Moreover, Tbps also exhibit low homology to other transport proteins and siderophore receptors. The homology among the members of this family of transporters suggests that the ancestral meningococcal Tf and Lf receptors may have been a single-unit transporter, similar to siderophore receptors (Perkins-Balding et al., 2004). The best characterized Tfbps and Lfbps to date belong to *Neisseria* species (Schryvers et al., 1998; Schryvers and Stojiljkovic, 1999).

### Production and Secretion of Siderophores/Hemophores

Siderophores and hemophores are relatively small (<1 kDa) compounds produced and secreted by some species of bacteria and fungi to acquire Fe. Once produced and secreted from microorganisms, their function is to chelate ferric Fe with very high affinity (formation constant up to 10^50^ M; Wandersman and Delepelaire, 2004; Saha et al., 2013). In fact, these compounds can remove ferric Fe from the host proteins Tf, Lf, and ferritin. Siderophores are produced as common products of microbial metabolism under Fe stress conditions and facilitate the solubilization of ferric Fe and transport Fe via a specific receptor expressed in the cell plasma membrane. In addition, *Bacillus subtilis* and *Mycobacterium smegmatis* that are unable to synthesize siderophores resolve this problem by using the Fe from siderophores derived from other microorganisms (xenosiderophores; Schumann and Mollmann, 2001; Miethke et al., 2013). The capacity of these systems to acquire Fe from various environmental or biological sources is an evident advantage for organisms that may exist in several niches. Hemophores, which are less well studied and only found thus far in Gram-negative bacteria, are employed via the following strategy: they are secreted into the extracellular medium where they scavenge heme from various hemoproteins due to their higher affinity for this compound and then return the heme to hemophore-specific outer membrane receptors (Wandersman and Delepelaire, 2012). These proteins are secreted under conditions of iron depletion (Faraldo-Gomez and Sansom, 2003; Wandersman and Delepelaire, 2012).

### Ferrireductases/Proteases

**FIGURE 2:**
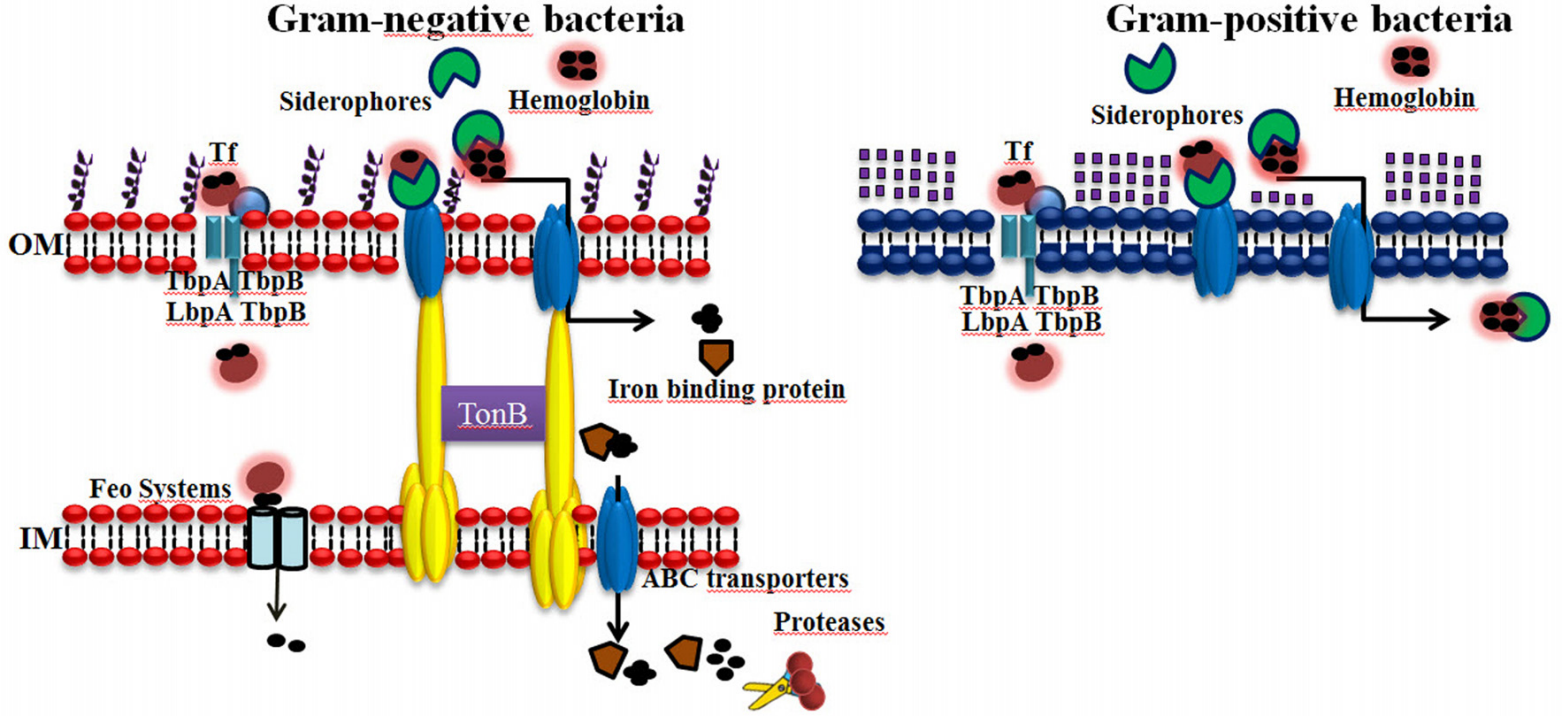
**Iron acquisition systems used by bacteria that infect humans.** Iron (Fe) is an essential element for virtually all forms of cellular life, including most bacteria, because it serves as a cofactor for several key enzymes required for many metabolic processes. Gram-negative and Gram-positive bacteria can acquire iron using elaborated mechanisms. (1) Receptors for host iron-containing proteins. (2) The production and secretion of siderophores/hemophores. (3) Ferrireductases/Proteases. The abilities of bacterial pathogens to adapt to the environment within a host are essential to their virulence.

Some microorganisms secrete ferrireductases or produce membrane-associated proteins that reduce the ferric iron in holoTf, holoLf, or ferritin to the more accessible ferrous form. The reduction of ferric iron destabilizes the host iron-containing protein, and the ferrous iron is thus released. It has been reported that some pathogens are able to produce and secrete proteases that cleave host iron-containing proteins, and iron is easily acquired by pathogens in this form, for example, *Entamoeba histoytica* has hemoglobinases (Payne, 1993; Serrano-Luna et al., 1998; Ortiz-Estrada et al., 2012). A scheme for Fe acquisition systems in bacteria is represented in **Figure 2**.

## Human Iron Sources and Iron Acquisition Systems in *V. parahaemolyticus*

*Vibrio parahaemolyticus* and other bacteria from the genus *Vibrio* including *V. cholerae, V. vulnificus*, require Fe for their growth and have developed systems during evolution to acquire Fe for sustaining metabolism and replication. As mentioned above, the Fe concentration in coastal waters ranges from 1.3–35.9 to 23.1–573.2 nM, which is sufficient for the growth of *V. parahaemolyticus*. In the human host, *V. parahemolyticus* can utilize host sources, such as the iron-proteins Hb, heme, hemin, and Tf (Yamamoto et al., 1994), and possibly others, such as Lf and Ft (**Figure 1**).

## Siderophores as a Mechanism of Iron Acquisition Used by *V. parahaemolyticus*

One of the strategies of *V. parahaemolyticus* to obtain the iron from the host iron-proteins is the use of siderophores. Under iron-limited conditions *V. parahaemolyticus* secretes Vibrioferrin to facilitate Fe acquisition. Fe-charged Vibrioferrin is recognized by an outer-membrane receptor composed of the proteins PvuA1 and PvuA2, which also recognizes heme and Hb. This receptor is coupled with the ABC transport system PvuBCDE which is located in the inner membrane and whose function is import the ferric-charged vibrioferrin to the inner membrane (Tanabe et al., 2003, 2011, 2012). *V. parahaemolyticus* also contains the TonB system which consists of three proteins designated TonB1, TonB2, TonB3 (Kuehl and Crosa, 2010). The energy for the transport of ferric-vibrioferrin is provided by the TonB2 system for PvuA1 and both the TonB1 and TonB2 systems for PvuA2 (Tanabe et al., 2011, 2012).

The *V. parahaemolyticus* also is able to utilize siderophores produced by other bacteria, for example, exogenous aerobactin, desferri-ferrichrome, and enterobactin (Funahashi et al., 2003, 2009; Tanabe et al., 2012). We have compared and analyzed *in silico* the mechanism used by *V. parahaemolyticus* with *V. cholerae*; the most studied member of the genus *Vibrio. V. cholerae* has multiple strategies for iron acquisition, including the endogenous siderophore vibriobactin and several siderophores that are produced by other microorganisms (Wyckoff et al., 2007). In general the *Vibrio* species, including *V. cholerae* and *V. anguillarum* can use catecholate-type siderophores as their cognate siderophores (Griffiths et al., 1984; Actis et al., 1986; Balado et al., 2009; Tanabe et al., 2012). Furthermore, some *Vibrio* sp. use the xenosiderophore enterobactin (Ent) that is produced mainly by members of the *Enterobacteriaceae* family (Griffiths et al., 1984; Naka and Crosa, 2012; Tanabe et al., 2012). On the other hand, Tan et al. (2014) described the use of the siderophore vulnibactin, essential in Fe uptake from host proteins. The importance of the vulnibactin in *V. vulnificus* pathogenicity was clinically demonstrated (Ceccarelli et al., 2013; Tan et al., 2014).

Once the iron has been acquired, *V. cholerae* has two systems for the transport of free iron: the Feo system, which transports ferrous iron, and the Fbp system, which transports ferric iron (Occhino et al., 1998; Wyckoff et al., 2007). It has been speculated that *V. cholerae* contains one additional high affinity iron transport system. Apparently, iron transport genes are regulated by Fur (Occhino et al., 1998; Wyckoff et al., 2007).

## Transferrin Receptors as an Iron Acquisition System Used by *V. parahaemolyticus*

Using a basic local alignment search tool (BLAST) analysis of the sequences in *V. parahaemolyticus*, we identified putative genes that could encode for Tf receptors, but not for Lf receptors. The putative *V. parahaemolyticus* Tf receptor gene (*tbpA*) had 88% of identity with for those reported for *N. meningitidis* (not shown; unpublished data). However, apparently *V. parahaemolyticus* also can use LF as in Fe source (Wong et al., 1996). This probably could be due to the siderophore utilization by this bacterium. Because of Lf is one of the main iron transporters at intestinal level, the *V. parahaemolyticus* capacity of to use Lf as an iron source and the mechanism from iron-Lf acquisition must to be determined. Other members of the genus *Vibrio* have receptors for the use of Tf as iron source. For example, recently; Pajuelo et al. (2015) demonstrated that pVvbt2 from *V. vulnificus*, which causes vibriosis in fish (mainly eels), encodes a host-specific Fe acquisition system that depends on an outer membrane receptor called Vep20. This protein recognizes eel Tf and belongs to a new family of plasmid-encoded fish-specific Tf receptors (Pajuelo et al., 2015). Furthermore, it was found that Vep20 is encoded by an iron-regulated gene that is overexpressed in eel blood during artificially induced vibriosis with *V. vulnificus* both *in vitro* and *in vivo* (Pajuelo et al., 2015). The *Vep20* gene homologs have been identified on the transferable plasmids of two species of fish pathogens with broad host ranges: *V. harveyi* (pVh1) and *Photobacterium damselae* subsp. *damselae* (pPHDD1; Pajuelo et al., 2015). It has been hypothesized that *V. cholerae* contains three proteins that could be Tf receptors. These proteins were shown to be involved in the binding of LF, Hemin, Ft, and Hb (Ascencio et al., 1992).

## Binding and Transport of Iron or Iron-Charged compounds in *V. parahaemolyticus*

**FIGURE 3:**
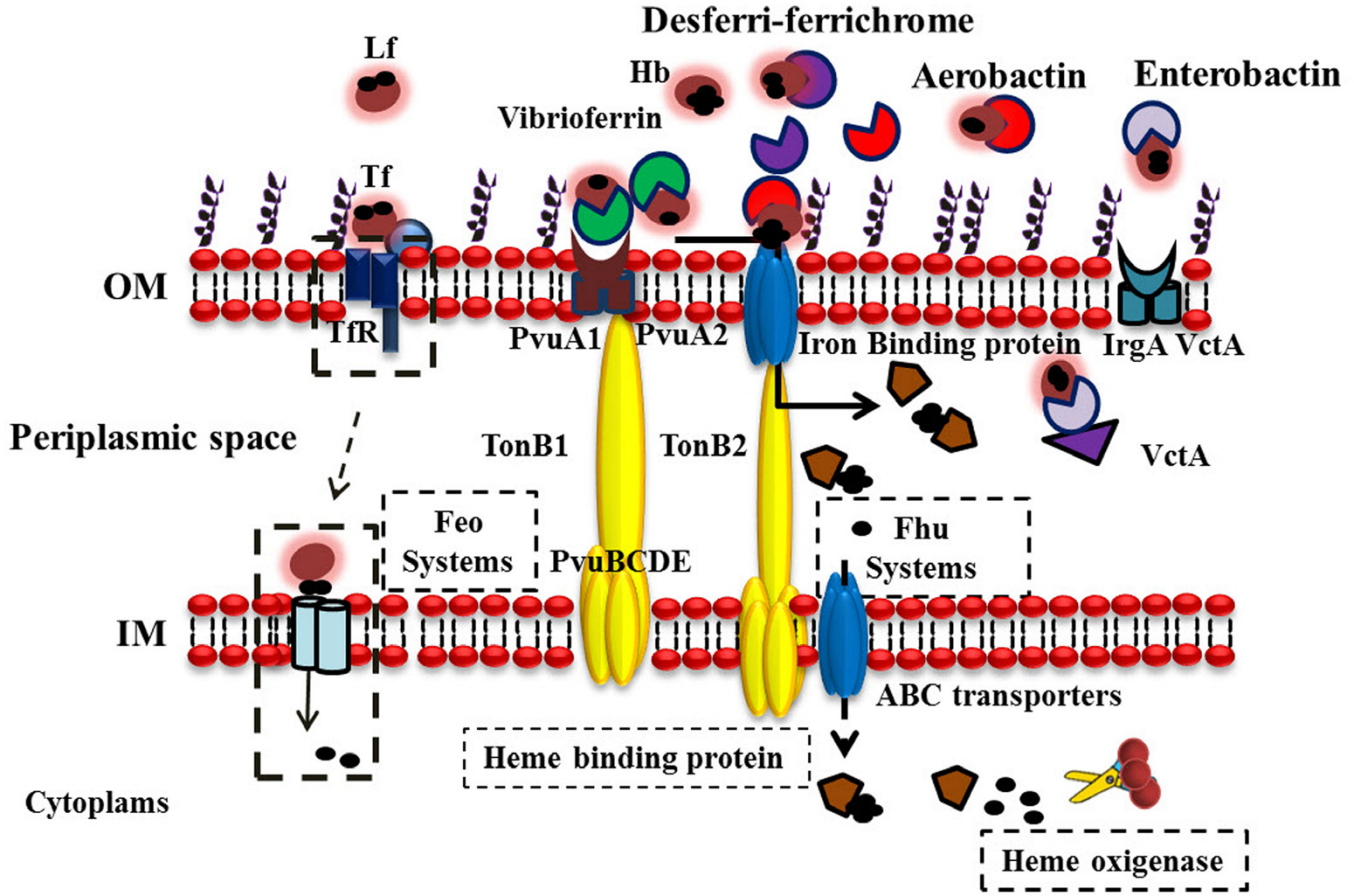
**Iron acquisition systems Used by *V. parahaemolyticus* to acquire iron**. *V. parahaemolyticus* can be found free swimming or attached to underwater surfaces, seafood products, and humans. The bacterium produces and chelates iron from different sources (depending on the habitat) and transports ferric-charged vibrioferrin into cells via the outer-membrane receptors PvuA1 and PvuA2 and the inner-membrane ABC transport system PvuBCDE. Three sets of TonB systems (TonB1, TonB2, and TonB3) are present in this bacterium, and the energy required for PvuA1 and PvuA2 to transport ferric vibrioferrin is provided by the TonB2 system for PvuA1 and both the TonB1 and TonB2 systems for PvuA2. *V. parahaemolyticus* can also utilize hydroxamate-type xenosiderophores, such as desferri-ferrichrome and aerobactin. Discontinuous lines indicate possible iron sources and iron acquisition mechanisms in *V. parahaemolyticus*.

The expression of two proteolytic proteins of 43 and 90 kDa from *V. parahaemolyticus* were identified. Apparently the protease of 43 kDa is capable of degrading Hb and it has been speculated that this could be one of the strategies of *V. parahaemolyticus* to acquire iron from the human host (Wong and Shyu, 1994). By using the BLAST, we also identified genes that could play a role in Fe acquisition (**Table 1**). Although they have not reported and their biological functions have not been described, these genes likely encode proteins involved in Fe acquisition for *V. parahaemolyticus* in different niches. The probable functions of putative Fe acquisition genes and homologies are described in **Table 1**. Additionally; a schematic view of Fe acquisition systems with putative proteins used by *V. parahaemolyticus* is shown in **Figure 3**.

**Table 1:** Genes related to Iron acquisition systems in *Vibrio cholerae* and putative genes in *Vibrio parahaemolyticus*.

*V. cholerae*			Homology *V. parahaemolyticus (%)*				
Protein/Gene	Function	Reference for source	Reference for source	Accession number	*Vibrio* sp.	Protein identity (%)	Gene identity (%)
VctA^∗^	Outer membrane receptor for enterobactin (VctA) Vct(PGD) participate in the transport of vibriobactin and enterobactin	Wyckoff et al. (2009)	Tanabe et al. (2012)	(CP006005.1)	*V. parahaemolyticus* O1:Kuk	65	67
VctP				(BA000032.2)	*V. parahaemolyticus* RIMD 2210633	56	66
VctG				(CP006005.1)	*V. parahaemolyticus* O1:Kuk	71	66
VctD				(BA000038.2)	*V. parahaemolyticus* RIMD 2210633	75	71
IrgA^∗^	Major membrane receptor for ferric enterobactin	Goldberg et al. (1990)		(CP001805.1)	*Vibrio* sp	69	69
TonB1^∗^	Energy transduction system, provides energy for transport of Enterobactin across the outer membrane	Occhino et al. (1998)	O’Malley et al. (1999)	(CP007005.1)	*V. parahaemolyticus* UCM-V493	51	70
TonB2^∗^	Energy transduction system, provides energy for the transport of vibrioferrin across the outer membrane	Wyckoff et al. (2004)		(CP006007.1)	*V. parahaemolyticus* 13-028/A3	54	72
FhuA^∗^	Transport of ferrichrome across the outer membrane	Rogers et al. (2000)	Funahashi et al. (2009)	(CP003973.1)	*V. parahaemolyticus* BB22OP	64	67
FhuB^∗^				(BA000032.2)	*V. parahaemolyticus* RIMD 2210633	77	70
FhuC*^∗^*				(AB300920.1)	*V. parahaemolyticus* W-9175	77	65
FhuD^∗^				(AB119276.1)	*V. parahaemolyticus*	62	69
HutA^∗^	Outer membrane receptors for heme and transporters	Mey and Payne (2001)	O’Malley et al. (1999)	(CP003973.1)	*V. parahaemolyticus* BB22OP	68	69
HutR				(CP006007.1)	*V. parahaemolyticus* 13-028/A3	54	67
HutB				(CP003973.1)	*V. parahaemolyticus* BB22OP	68	64
HutC^∗^			O’Malley et al. (1999)	(CP006008.1)	*V. parahaemolyticus* O1:K33	67	70
HutD				(CP006008.1)	*V. parahaemolyticus* O1:K33	68	68
FeoA	Ferrous iron transporter	Cartron et al. (2006)		(CP007004.1)	*V. parahaemolyticus* UCM-V493	73	68
FeoB				(CP006004.1)	*V. parahaemolyticus* O1:Kuk	79	73
FeoC				-	*V. parahaemolyticus*	51	-
FbpA	Ferric iron transporter	Kirby et al. (1998)		(CP006008.1)	*V. parahaemolyticus* O1:K33	81	75
FbpB	Periplasmic ferric iron binding protein			(BA000031.1)	*V. parahaemolyticus*	78	70

*indicate proteins that were studied and reported in V. parahaemoyticus.

*Column 6 contain the V. parahaemolyticus strain with the highest identity.*

*FeoC was not found in the V. parahaemolyticus strains.*

Apparently, the *V. parahaemolyticus* contain the proteins VctA, VctP, VctG, and VctD (Tanabe et al., 2012). These proteins are involved in the use of the siderophore ferric-enterobactin (Wyckoff et al., 2007, 2009). VctA and IrgA are receptors for enterobactin in *V. cholerae* and *V. parahaemolyticus* (Goldberg et al., 1990; Tanabe et al., 2012). Also, in *V. parahaemolyticus* were identified the TonB1 and TonB2 system similar to *V. cholerae* (Occhino et al., 1998; Seliger et al., 2001; Wyckoff et al., 2004). The proteins that belong to the TonB systems are involved in the transport of Fe^3+^ in Gram-negative bacteria. The system includes outer membrane receptors, all of which are connected with a complex of proteins located at the inner membrane such as TonB, ExbB, and ExbD. This system also includes a periplasmic binding protein associated with an ABC transporter (Larsen et al., 1996; Koster, 2005). The first step is the binding of the ferric-siderophore to the receptor, it has been speculated that this binding induces a conformational change and then the interaction of the receptor with TonB is enhanced. The proteins ExbB and ExbD provide the energy for the transport of the ferric-siderophore to the inner membrane, in this second step a periplasmic binding protein associated with an ABC transporter delivers the ferric-siderophore into the cytoplasm, in the third step the Fe dissociates from the siderophore (Larsen et al., 1996; Occhino et al., 1998; O’Malley et al., 1999). Little is known about the recycling, storage, and modification of the siderophore. The secretion of *E. coli* enterobactin is mediated by the membrane exporter protein EntS (Furrer et al., 2002; Danese et al., 2004).

In *V. parahaemolyticus* the genes encoding both for the TonB1 and TonB2 systems are located on the small chromosome, and the TonB3 system on the large chromosome (O’Malley et al., 1999; Kuehl and Crosa, 2010; Tanabe et al., 2012). In *V. parahaemolyticus* TonB2 is most active than TonB1 in providing the energy necessary for the transport of ferric-enterobactin via the receptors IrgA and VctA (Tanabe et al., 2012). However, this is different in *V. cholerae*, during the transport of ferric-enterobactin the energy required for IrgA and VctA receptors is provided by the TonB2 system (Seliger et al., 2001; Tanabe et al., 2012). The TonB3 system is not implicated in the transport of iron either *V. parahaemolyticus* or *V. cholerae* (Kuehl and Crosa, 2010; Tanabe et al., 2012), has been reported that the TonB3 system from *V. vulnificus* is induced when the bacterium grows in human serum (Alice and Crosa, 2012). The *V. cholerae* has two TonB systems, which are present on small chromosome (this is different from *V. parahaemolyticus*), and those encoding the TonB2 system are located on the large chromosome. They have unique as well as common functions (Seliger et al., 2001). Both mediate the transport of hemin, vibriobactin, and ferrichrome. However, only TonB1 participates in the use of the siderophore schizokinen, but TonB2 is required for the transport of enterobactin (Seliger et al., 2001).

With respect to FhuA, this protein is the receptor for the siderophores desferri-ferrichrome and aerobactin in *V. parahaemolyticus* (Funahashi et al., 2009). In addition, FhuB, FhuC, and FhuD apparently are involved in the transport of the siderophores, and also are present in *V. cholerae* (Rogers et al., 2000; Funahashi et al., 2009). Regarding the hut genes, it has been reported that HutA is the receptor for the uptake of heme. In addition, HutR has significant homology to HutA as well as to other outer membrane heme receptors (Occhino et al., 1998; Mey and Payne, 2001). In the *V. cholerae* the presence of hutBCD stimulated growth when hemin was the iron source, but these genes were not essential for hemin utilization (Occhino et al., 1998; Mey and Payne, 2001; Wyckoff et al., 2004). Other genes found in the *V. parahaemolyticus* genome were the feo system. The feo system consists of genes that encoded proteins involved in the transport of ferrous iron (Fe^2+^), which is expected to be a major iron source in the intestine (Cartron et al., 2006). This Fe^2+^ iron transport system feo is widely distributed among bacterial species such as *V. cholerae.* In this bacterium, the feo operon consists of three genes, feoABC. FeoB is an 83-kDa protein involving in the pore formation for iron transport (Weaver et al., 2013). FeoA and FeoC are all required for iron acquisition; however, their functions have not been described in detail. Apparently, in the genome of *V. parahaemolyticus* there are genes that encode for this Fe^2+^ transport. Moreover, the *V. parahaemolyticus* contains other iron transporters such as FbpA and FbpB. In *Pasteurella haemolytica* the presence of FbpABC family of iron uptake systems has been documented (Kirby et al., 1998). This family of proteins is involved in the utilization and transport of the ferric-xenosiderophore of the bacterium *N. gonorrhoeae*, and is independent of the TonB system (Strange et al., 2011). We speculate that *V. parahaemolyticus* bacteria could have this family of proteins in order to acquire ferric iron from xenosiderophores, in a TonB-independient manner. All of these Fe acquisition systems could be likely involved in the survival of *V. parahaemolyticus* and other *Vibrio* sp. in the different environments that they can colonize, i.e., water, humans, and several other vertebrate hosts (Fouz et al., 1997).

## Conclusion

The element Fe is essential for the growth of pathogenic microorganisms, is fundamental and necessary for establishment and replication inside a host, and is required to cause infection. To this end, microbes that live in hostile environments and extracellular spaces of their host must employ different strategies for Fe acquisition to be successful in these niches. It has been postulated that such strategies were acquired during evolution and are involved in the pathogenesis and virulence of bacteria such as *V. parahaemolyticus*. Based on reported findings, this bacterium can utilize the Fe from the proteins Tf, Hb, and hemin by means of siderophores (vibrioferrin, aerobactin, and desferri-ferrichrome) and likely also receptors to acquire Fe from humans during infection of the gut. In low iron, *V. parahaemolyticus* express two proteins of 78 and 83 kDa (now called PvuA2 and PvuA1; respectively), which are the receptors for the siderophore vibrioferrin, and a protease of 43 kDa, which has been hypothesized is involved in one of the strategies of *V. parahaemolyticus* in order to acquire iron from the host. *V. parahaemolyticus* also encodes for LutA, which is the receptor for the siderophore aerobactin. Additionally, according to **Table 1** and other works, this pathogen possesses genes that encode accessory proteins involved in Fe acquisition, transport and synthesis of molecules implicated in Fe acquisition systems.

The iron *per se* has been involved in increase the virulence of *V. parahaemolyticus* and other bacteria. For example, in recent works it have been demonstrated that iron uptake and Quorum sensing (QS) can act together as global regulators of bacterial virulence factors (Wen et al., 2012). QS is a regulatory mechanism used by several bacteria to regulate or modulate the production of extracellular compounds at high cell densities with the aim of establish bacterial biofilms (nowadays, the main medical problem for the control of infectious diseases). Bacterial QS serves as simple indicator of population density, by means of secreting signaling molecules called autoinducers. The link among iron and QS was reported firstly in *Pseudomonas aeuruginos*a (Bollinger et al., 2001). This bacterium in iron-depleted conditions, retarded biofilm formation and increased the twitching motility and expression of QS-related genes, suggesting a link between iron and QS system during biofilm formation (the most important virulence factor of *P. aeruginosa;* Cai et al., 2010), in contrast; *Staphylococcus aureus* in Fe limitation appeared to stimulate biofilm formation (Johnson et al., 2005). These controversial observations can be explained because biofilm formation QS-dependent is nutritionally conditional (Shrout et al., 2006). In other words, in the absence of an acquisition system needed for obtain nutritional iron, or other nutrients such as carbon sources, a bacterium such as *P. aeuruginosa* could establish thin and weak biofilms instead of mature biofilms (Banin et al., 2005; Shrout et al., 2006). At intracellular level the main regulator is Fur. Therefore, Fur regulates genes that are crucial for the iron acquisition needed for the bacteria replication and consequently; in biofilms development (Banin et al., 2005).

Until now, the association between iron and QS on biofilms formed by *V. parahaemolyticus* has not been studied. The results of a BLAST search indicate that some strains of V. *parahaemolyticus* have genes involved in QS and biofilm formation (luxP, luxQ, cqsA, luxO, hapR, aphA), with high identity for those reported in the *V. cholerae* (Jobling and Holmes, 1997; Skorupski and Taylor, 1999; Miller et al., 2002; Zhu and Mekalanos, 2003; Raychaudhuri et al., 2006; **Table 2**). We speculate that iron and QS could be involved in the virulence of *V. parahaemolyticus*. In the *V. vulnificus* the biosynthesis of the siderophore vulnibactin is regulated by Fur and QS (Wen et al., 2012; Kim et al., 2013). Once vulnibactin sequesters the iron needed for the replication of the bacterium, *V. vulnificus* catalizes the enzyme LuxS, which synthetizes the autoinducer (AI-2), involved in the activation of signals for QS and Biofilm formation. At high cell density, *V. vulnificus* enhances the expression of the gene *vvpE*, which encodes for the virulence factor elastase (Kim et al., 2013). It has been observed that mutations in LuxS reduce not only biofilm formation, also reduce virulence factors such as motility, production of proteases and the secretion of the *V. vulnificus* hemolysin, etc. (Wen et al., 2012).

**Table 2:** Genes related to quorum sensing in *Vibrio cholerae* and putative genes in *Vibrio parahaemolyticus*.

*V. cholerae*			*V. parahaemolyticus*			
Protein/Gen	Function	Reference	Accession number	*Vibrio* sp.	Protein identity	Gene identity
luxP	Detects the AI-2 as quorum sensing (QS) signal.	Miller et al. (2002)	CP006007.1	*V. parahaemolyticus* O1:K33	65%	67%
luxQ	Can be autophosphorylated, resulting in the transfer of a phosphate group to LuxO.	Raychaudhuri et al. (2006)	CP006005.1	*V. parahaemolyticus* O1:Kuk	-	64%
cqsA	Acts as an autoinducer to form biofilms.	Zhu and Mekalanos (2003)	BA000032.2	*V. parahaemolyticus* RIMD 2210633	59%	74%
luxO	Activates expression of four sRNAs that destabilize hapR mRNA repressing expression of HapR.	Jobling and Holmes (1997)	CP007004.1	*V. parahaemolyticus* UCM-V493	-	75%
hapR	Master regulator of QS.	Jobling and Holmes (1997)	CP006008.1	*V. parahaemolyticus* O1:K33	72%	75%
aphA	Is a winged-helix transcription factor that controls virulence factor production in the closely related pathogen and QS.	Jobling and Holmes (1997), Skorupski and Taylor (1999)	CP007004.1	*V. parahaemolyticus UCM-V493*		68%

Although the link between Fur and QS is complex, the siderophores production and the coordinated regulation by the two systems (Fur and QS) probably ensures to bacteria maintain an appropriate iron concentration to optimize its survival and propagation within the human host (Wen et al., 2012). It has been shown that blocking the nutritional support and the communication pathways of one’s adversaries serves as an effective tactic to disrupt cooperative actions among individuals or groups. Removal of iron as a therapeutic approach has been investigated *in vitro* for several infections, with promising results (Gorska et al., 2014). The generation of analogs that block or alternative signals involved in QS have been developed, in order to disrupt biofilm formation and other virulence factors (LaSarre and Federle, 2013). These strategies could be success in bacteria, because Fe limitation and Fe excess affect QS-dependent biofilm formation, therefore understand how these sophisticated and complex regulatory systems are regulated, is vital to predict bacterial behaviors and possibly then, develop drugs that can interfere with the iron acquisitions systems, or with the response of signal molecules involved in iron acquisition systems or QS (Bollinger et al., 2001; Wen et al., 2012; LaSarre and Federle, 2013).

When the host tries to limit infection by lowering iron, pathogens such as *V. parahaemolyticus* triggered increased expression of virulence factors (that are relevant for surface colonization and infection) in order to cause damage to the host. Based in these results, we conclude that unnecessary or excessive iron administration may be harmful, due the possible multiplication of bacterial growth and increase in their virulence. While it is clear that iron levels are important in infection, it is not an easy task to control their levels in the host. The complete detailed mechanism for Fe acquisition and its role in *V. parahaemolyticus* virulence remains to be determined.

## Conflict of Interest Statement

The authors declare that the research was conducted in the absence of any commercial or financial relationships that could be construed as a potential conflict of interest.
